# Nasal Bone Shape Is under Complex Epistatic Genetic Control in Mouse Interspecific Recombinant Congenic Strains

**DOI:** 10.1371/journal.pone.0037721

**Published:** 2012-05-25

**Authors:** Gaétan Burgio, Michel Baylac, Evelyne Heyer, Xavier Montagutelli

**Affiliations:** 1 Institut Pasteur, Unité de Génétique Fonctionnelle de la Souris, Centre National de la Recherche Scientifique, URA 2578, Paris, France; 2 Unité d’Eco-Anthropologie, équipe, Génétique des Populations Humaines, Centre National de la Recherche Scientifique, Museum National d’Histoire Naturelle, Université Paris 7, UMR 7206, Paris, France; 3 Department of Genetics, Menzies Research Institute, University of Tasmania, Hobart, Tasmania, Australia; 4 Organisation, Structure et Evolution de la Biodiversité UMR 5202 et Plateforme de Morphométrie, Centre National de la Recherche Scientifique IFR 101 et UMR 5202, Laboratoire d’Entomologie du Muséum National d’Histoire Naturelle, Paris, France; Karolinska Institutet, Sweden

## Abstract

**Background:**

Genetic determinism of cranial morphology in the mouse is still largely unknown, despite the localization of putative QTLs and the identification of genes associated with Mendelian skull malformations. To approach the dissection of this multigenic control, we have used a set of interspecific recombinant congenic strains (IRCS) produced between C57BL/6 and mice of the distant species *Mus spretus* (SEG/Pas). Each strain has inherited 1.3% of its genome from SEG/Pas under the form of few, small-sized, chromosomal segments.

**Results:**

The shape of the nasal bone was studied using outline analysis combined with Fourier descriptors, and differential features were identified between IRCS BcG-66H and C57BL/6. An F2 cross between BcG-66H and C57BL/6 revealed that, out of the three SEG/Pas-derived chromosomal regions present in BcG-66H, two were involved. Segments on chromosomes 1 (∼32 Mb) and 18 (∼13 Mb) showed additive effect on nasal bone shape. The three chromosomal regions present in BcG-66H were isolated in congenic strains to study their individual effect. Epistatic interactions were assessed in bicongenic strains.

**Conclusions:**

Our results show that, besides a strong individual effect, the QTL on chromosome 1 interacts with genes on chromosomes 13 and 18. This study demonstrates that nasal bone shape is under complex genetic control but can be efficiently dissected in the mouse using appropriate genetic tools and shape descriptors.

## Introduction

The skull is a complex three-dimensional structure, with features highly adapted to specialized functions. Its shape results from the action of genes involved in finely tuned developmental processes. The resemblance of monozygotic twins suggests that skull shape is under tight genetic control. Besides, dramatic variations in craniofacial morphology among individuals of a given species, ranging from subtle changes to profound differences, result from genetic factors which number, nature and function remain largely elusive. Identification of underlying genes is challenging, but could provide an efficient approach to better understand the formation of flat bones in connection with the surrounding soft tissues.

Several groups have attempted to tackle the complexity of skull shape in mice using different approaches. While variations in natural or pedigreed populations have been used for example in plants [1] fishes [2] or primates [3], studies in mice have been made on either F2 progeny [4], or recombinant inbred strains [5], using classical measurements [6], or geometric morphometrics [7], [8]. Compared with segregating populations such as F2s, where every individual carries a unique genotype, recombinant inbred strains allow for replications, since a trait can be measured on a group of genetically identical, sex- and age-matched individuals, buffering between-individual noise. This results in more power for both the identification and precise localization of QTLs. However, published studies have failed so far to provide small-sized confidence intervals amenable to the positional cloning of underlying genes.

Recombinant congenic strains have been developed as a tool to dissect polygenic traits [9]. Each strain carries 12.5% of its genome from a donor strain, and 87.5% from a recipient strain. In fact, recombinant congenic strains have proven efficient to identify genetic factors for various traits [10], [11], [12], including their epistatic interactions [13]. While all existing sets have been developed between laboratory inbred strains, we have combined this strategy with the very high polymorphism rate inherent to interspecific crosses. As a result, we have produced a series of 55 Interspecific Recombinant Congenic Strains (IRCS) with the *Mus spretus*-derived SEG/Pas (SEG) strain as a donor, and C57BL/6J (B6) as a recipient strain. Genotyping has revealed that each strain of the collection inherited on average only 1.3% of its genome from SEG, under the form of a few chromosomal segments with an average size of 13****Mb [14]. We have used this collection to study the genetic determinism of variations in the shape of the nasal bone. This most rostral part of the skull is likely under less biomechanical constraint, hence may show more variations. In addition, being almost flat, the nasal bone can be easily studied by outline analysis. The characterization of one of the IRCS led to the identification of three QTLs with strong epistatic interactions. Since this strain includes only a fraction of the phenotypic difference between B6 and SEG, our results suggest that the nasal bone shape is controlled by more than a few genes, under complex genetic interplay.

## Results

### Nasal Bone Shape

**Figure 1:**
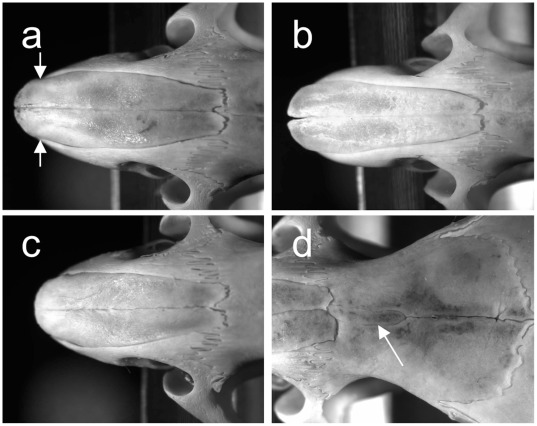
Dorsal view of the rostral part of the skull showing the nasal bone of 60±5 days old male mice. A: C57BL/6 (arrows show the rostral depression); B: SEG (*Mus spretus*); C: IRCS strain 66H; D: caudal region of the nasal bone in C57BL/6 showing the interfrontal bone (arrow).

**Figure 2:**
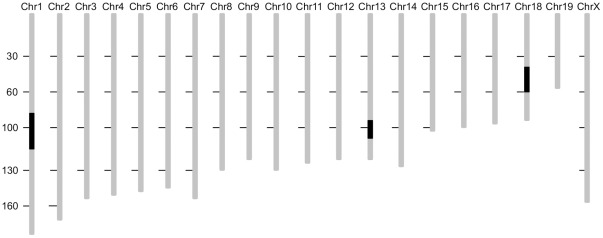
Genetic map of 66H IRCS indicating the position and sizes of the SEG-derived segments. The segments of *Mus spretus* origin are displayed in solid while B6 segments are shaded. The 66H strain contains three SEG-derived segments on the Chromosome 1, 13 and 18.

Nineteen IRCS strains were compared to B6 and SEG/Pas strain for global skull shape. At least 15 age-matched male were analyzed for each strain. Macroscopic comparison of SEG/Pas and B6 revealed marked differences in the shape of the nasal bone (Figure 1A, and 1B). In B6, the rostral and caudal ends have the same size, giving to the bone a rectangular shape. The caudal end shows a notch in relation with the interfrontal bone (Figure 1D), and the lateral sides show a depression towards the rostral end (Figure 1A). In SEG/Pas, the nasal bone has a trapezoidal outline (Figure 1B). The caudal notch is vestigial, the interfrontal bone is absent, and the rostral end has a round shape with no depression. Among the IRCS examined, BcG-66H (66H thereafter) stood out with features intermediate between those of the two parental strains (Figure 1C). The caudal notch is deeper than in B6, while interfrontal bone is absent. The rostral part is rounded, as in SEG/Pas. 66H was chosen for genetic analysis (Figure 2) using F1 and F2 hybrids, congenic and bicongenic strains. To allow for QTL mapping, nasal bone shape was submitted to outline analysis to describe the shapes with quantitative variables.

### Conditions for Outline Analysis

The three main parameters of the outline analysis are the number of points describing the outline, the number of harmonics used to fit the points, and of the number of principal component analysis (PCA) axes retained to reduce data dimensionality. Using our experimental data on 868 outlines, we estimated the optimal values for these parameters. We found that 100 points and 30 harmonics were necessary to describe the details of the nasal bone outlines (Figure S1), and fifteen PCA axes included 99% of the total variance. All subsequent analyses were performed under these conditions.

### Outline Analysis of Nasal Bone in B6, 66H and (B6 × 66H)F1

**Figure 3:**
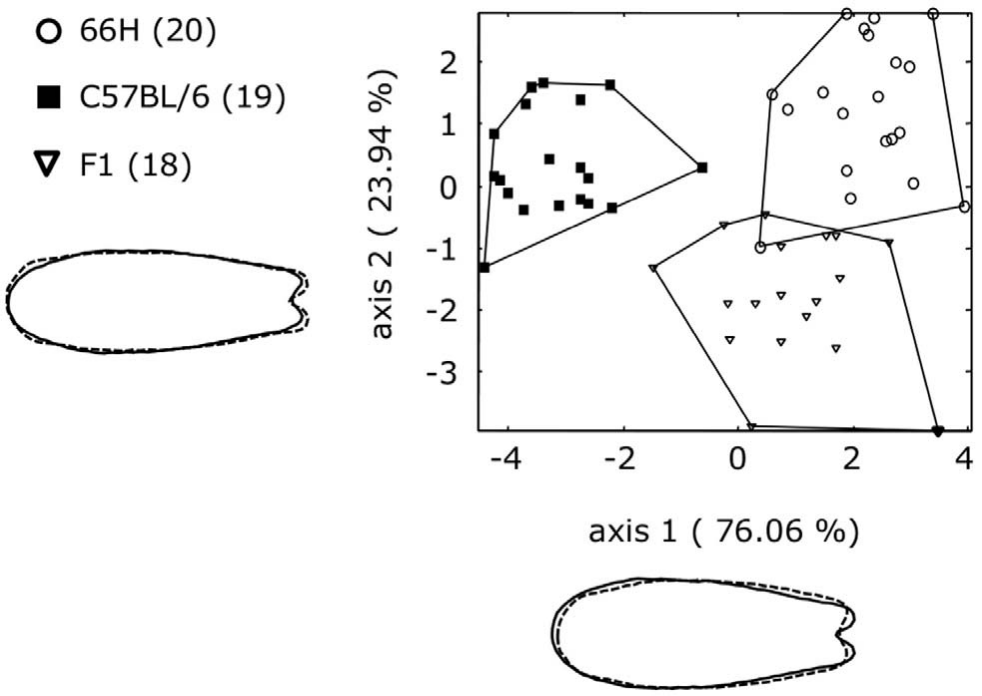
Comparison of nasal bone shape in B6, 66H and their F1 hybrids by linear discriminant analysis (LDA) based on 15 principal components axes on a combination of Procrustes superimposition and elliptic Fourier descriptors (30 harmonics). The first and second canonical axes were represented. The number of mice in each group is given in parentheses. Shapes drawn outside the graph describe nasal bone shape variations associated with low values (dashed line) or high values (solid line) along the axes. B6 and 66H fall into two well separated groups. F1 hybrids are distinct from either parent. Shape drawn outside the scatterplot, calculated with a multivariate regression, describes nasal bone shape variation along the canonical axes with low values (in dashed lines) and high values (in solid lines). No amplification and the nasal bone shape changes was effected.

Eighteen to twenty males from each strain or F1 were submitted to nasal bone outline analysis. Shape differences between strains B6 and 66H were assessed by linear discriminant analysis on Fourier shape space. Figure 3 shows the result on B6, 66H and F1 mice for the two canonical axes. The first axis (76.1% of total variance) separates clearly B6 and 66H. The second axis (23.9% of total variance) contrasts F1 with B6 and 66H. Representations of extreme shapes along the two axes confirmed macroscopic observations, with deeper caudal notch associated with higher values along the first axis (66H and F1 greater than B6) and with lower values along the second axis (F1 more than 66H and B6). Leave-one-out cross validation percentage reached 84.2%, with three out of 18 F1 mice misclassified as 66H. Statistical inference on Mahalanobis distances showed that B6 was significantly different from both 66H (T^2^ = 91, F*_(3,38)_* = 629, p = 0.005) and F1 (T^2^ = 62.6, F*_(3,46)_* = 356, p = 0.008). Differences between 66H and F1 were also significant (T^2^ = 33, F*_(3,47)_* = 138, p = 0.021).

### QTL Mapping on (66H × B6)F2 Mice

To identify which of the three SEG-derived chromosomal regions present in 66H were responsible for nasal bone shape difference, 91 (66H × B6)F2 male mice were produced and analyzed. For QTL mapping, the phenotypic value of F2 mice was calculated by applying to the nasal bone measurements the canonical coefficients calculated from the LDA analysis of B6 and 66H. This resulted in a normal distribution of phenotypes covering the range of the two parental strains (Figure S2). Single marker ANOVA revealed highly significant association with genotype at Chr 1 and Chr 18 markers (peaks at *D1Mit306* and *D18Mit123*, respectively, Table 1). This result was confirmed using R/QTL (LOD score of 1.99 for *D1Mit306*, and 3.18 for *D18Mit123*, significant threshold at 5% = 1.89). For both markers, SEG allele was associated with a decreased phenotypic value, as anticipated from the parental strains (B6∶3.28±0.20; SEG: -3.12±0.18), and heterozygotes were intermediate between B6/B6 and SEG/SEG homozygotes, suggesting codominant effect.

**Table 1:** Effect of Chr 1, Chr 13, and Chr 18 markers on LDA score in (66H x B6)F2 mice (N = 91).

			LDA score (mean±s.e.m.)			
	Chr	Position	B/B	B/S	S/S	p-value
*D1Mit84*	1	93.7 Mb	1.48±0.32	1.08±0.53	−0.2±0.53	0.03
*D1Mit306*	1	98.7 Mb	1.99±0.38	0.63±0.41	−0.06±0.54	0.0064
*D13Mit290*	13	103.9 Mb	0.34±0.44	1.41±0.38	0.95±0.57	0.24
*D18Mit123*	18	42.7 Mb	2.48±0.66	1.03±0.3	−0.25±0.46	0.00078
*D18Mit58*	18	56 Mb	2.48±0.66	0.85±0.32	−0.04±0.46	0.003

**Figure 4:**
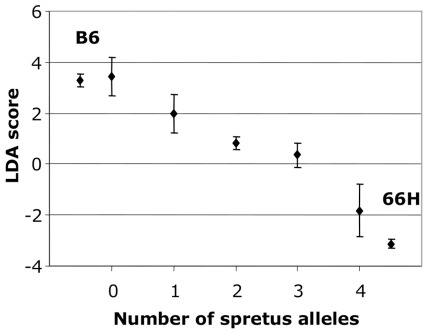
Cumulative effect of Chr1 and Chr18 QTLs on nasal bone shape. The projection on the canonical axis calculated as a LDA score (see text) is plotted against the number of SEG alleles inherited by the F2 progeny at both Chr1 and Chr18 QTLs. Parental strains are shown at extreme positions. Error bars represent s.e.m. The scores in F2 progeny decrease as a function of the number of SEG alleles and encompass the difference between 66H and B6 parental strains.

Two-way ANOVA between pairwise combinations of markers did not reveal epistatic interactions between the three chromosomal regions. Phenotypic values of F2 mice decreased almost linearly with the total number of SEG alleles carried at *D1Mit306* and *D18Mit123* (Figure 4) suggesting that the two loci act additively. Mice with four B6 alleles had a phenotype close to that of B6 mice, while mice with four SEG alleles, were phenotypically close to 66H. From these results, we concluded that the Chr 1 and Chr 18 regions were sufficient to explain the phenotypic differences between B6 and 66H.

### Analysis of Congenic Strain

**Figure 5:**
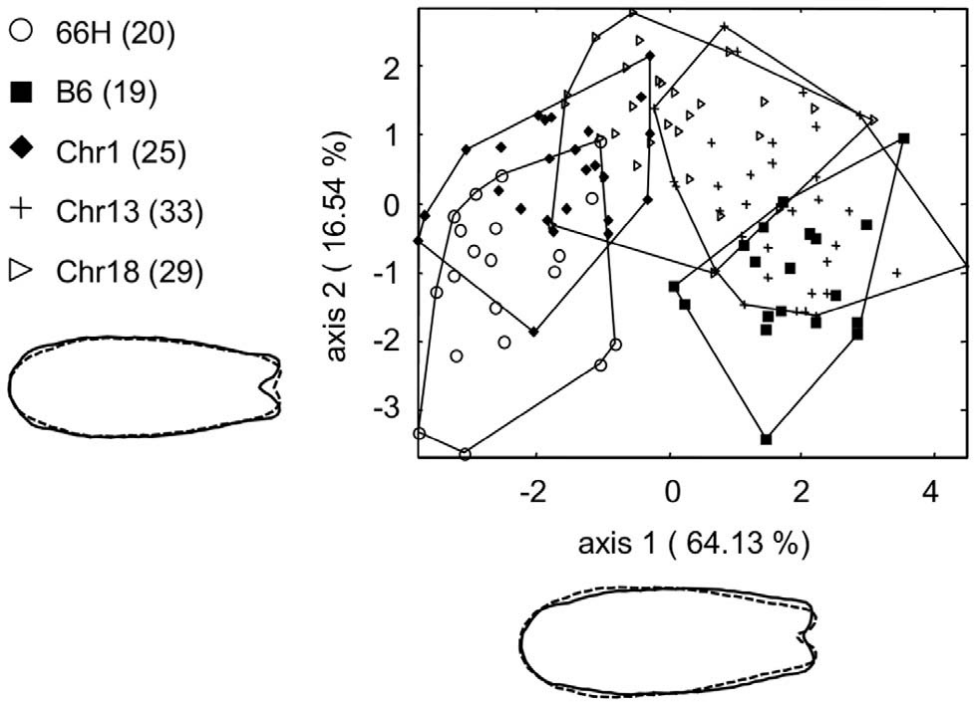
Comparison of nasal bone shape in B6, 66H and in Chr1, Chr13, and Chr18 congenic mice, by LDA based on 15 principal components axes on a combination of Procrustes superimposition and elliptic Fourier descriptors (30 harmonics). The first two canonical axes are represented, totaling 80.67% of total variance. Chr1 congenics are close to 66H, while Chr13 congenics partially overlap with B6. The shape of Chr18 congenics is intermediate between that of the two parental strains.

Since genotype to phenotype correlations are more efficiently studied in groups of genetically homogeneous individuals, each SEG-derived chromosomal region present in 66H was introduced by two backcrosses into a congenic strain (designated B6-Chr1, B6-Chr13, and B6-Chr18). Twenty-five to 33 mice per congenic strain were analyzed. Figure 5 shows the first two canonical axes of LDA performed on the three congenic strains, B6, and 66H. On the first axis (64.13% of total variance), B6-Chr1 and 66H are separated from B6 and B6-Chr13, while B6-Chr18 spreads across the two groups. Visualisation of shape changes along this axis is consistent with previous observations and emphasizes variations in bone width in the rostral and caudal regions, resulting in rectangular versus trapezoidal shape. The second axis (16.54%) introduces separation between B6-Chr18 and parental strains. Visualisation of shape changes underlines the wide caudal part, deep and opened caudal notch, and large, rounded rostral part of the nasal bone in B6-Chr18.

Statistical inference on Mahalanobis distances confirmed that B6-Chr1 did not quite significantly differ from 66H (T^2^ = 11.7, F*_(5,40)_* = 18.24, p = 0.06 with 1-β = 0.94), neither did B6-Chr13 from B6 (T^2^ = 11.5, F*_(5,45)_* = 11.5, p = 0.06 with 1-β  = 0.9). Conversely, B6-Chr18 was different from all other strains (B6-Chr1: T^2^ = 17.6, F*_(5,53)_* = 30.04, p = 0.021; B6-Chr13: T^2^ = 22.15, F*_(5,58)_* = 39.7, p = 0.012; B6: T^2^ = 19.6, F*_(5,44)_* = 34.08, p = 0.017; 66H: T^2^ = 26.3, F*_(5,45)_* = 49.05, p = 0.008). These results confirmed the individual effects of Chr 1 and Chr 18 QTLs observed in the F2 cross.

**Figure 6:**
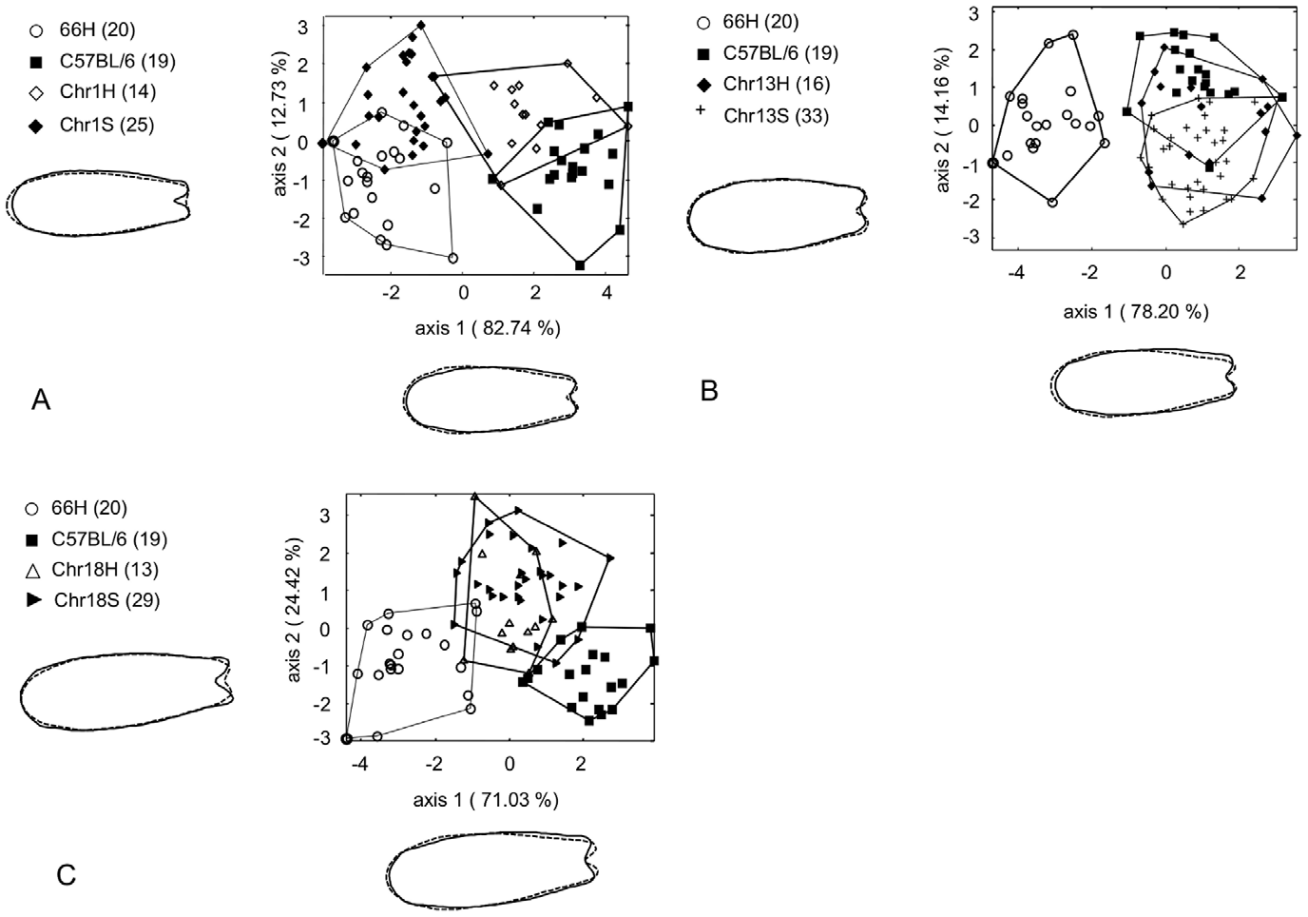
Plots of the first and second canonical axes from LDA with elliptic Fourier descriptor on nasal bone shape to assess the mode of inheritance of the three chromosomal regions. A: The first and second canonical axes for 66H, B6, Chr1 (Chr1 congenics), and Chr1H (heterozygotes for Chr1) are represented, totaling 95.5% of the total variation and showed the same phenotype between 66H and Chr1S whereas Chr1H exhibited a shape difference with B6 and Chr1S. Therefore Chr 1 QTL is semi-dominant. B: the first and second canonical axes of 66H, B6, Chr13 (Chr13 congenics), Chr13H (heterozygotes for Chr13) accounted for 92.4% of the total shape variation displayed no specific inheritance pattern. Neither heterozygotes nor homozygotes for Chr13 are different from B6 C: the first and second canonical axes of 66H, B6, Chr18 (Chr18 congenics), Chr18H (heterozygotes for Chr18) represented 95.4% of the total variance exhibited a similar intermediate shape between 66H and B6 for Chr18H and Chr18S. Therefore Chr18 QTL is dominantly inherited. For the explanation of the shape changes, see Figure 3. Shape changes were not amplified.

In addition, we analysed several heterozygous individuals for each congenic strain. We found that heterozygous B6-Chr1 did not differ from B6 (T^2^ = 9.4, F*_(4,32)_* = 16, p = 0.12), suggesting that, in contrast with results from the F2 cross, the SEG allele at Chr 1 locus was recessive (Figure 6). It was dominant at the Chr 18 locus since there was no difference between heterozygous and homozygous B6-Chr18 mice (T^2^ = 4, F*_(4,40)_* = 5.77, p = 0.5). Both heterozygous and homozygous B6-Chr13 mice were undistinguishable from B6 (respectively T^2^ = 6.55, F*_(4,33)_* = 10.1, p = 0.23 and T^2^ = 12, F*_(4,50)_* = 24, p = 0.07 with 1-β = 0.87).

### Analysis of Bicongenic Strains Reveal Strong Epistasis

**Figure 7:**
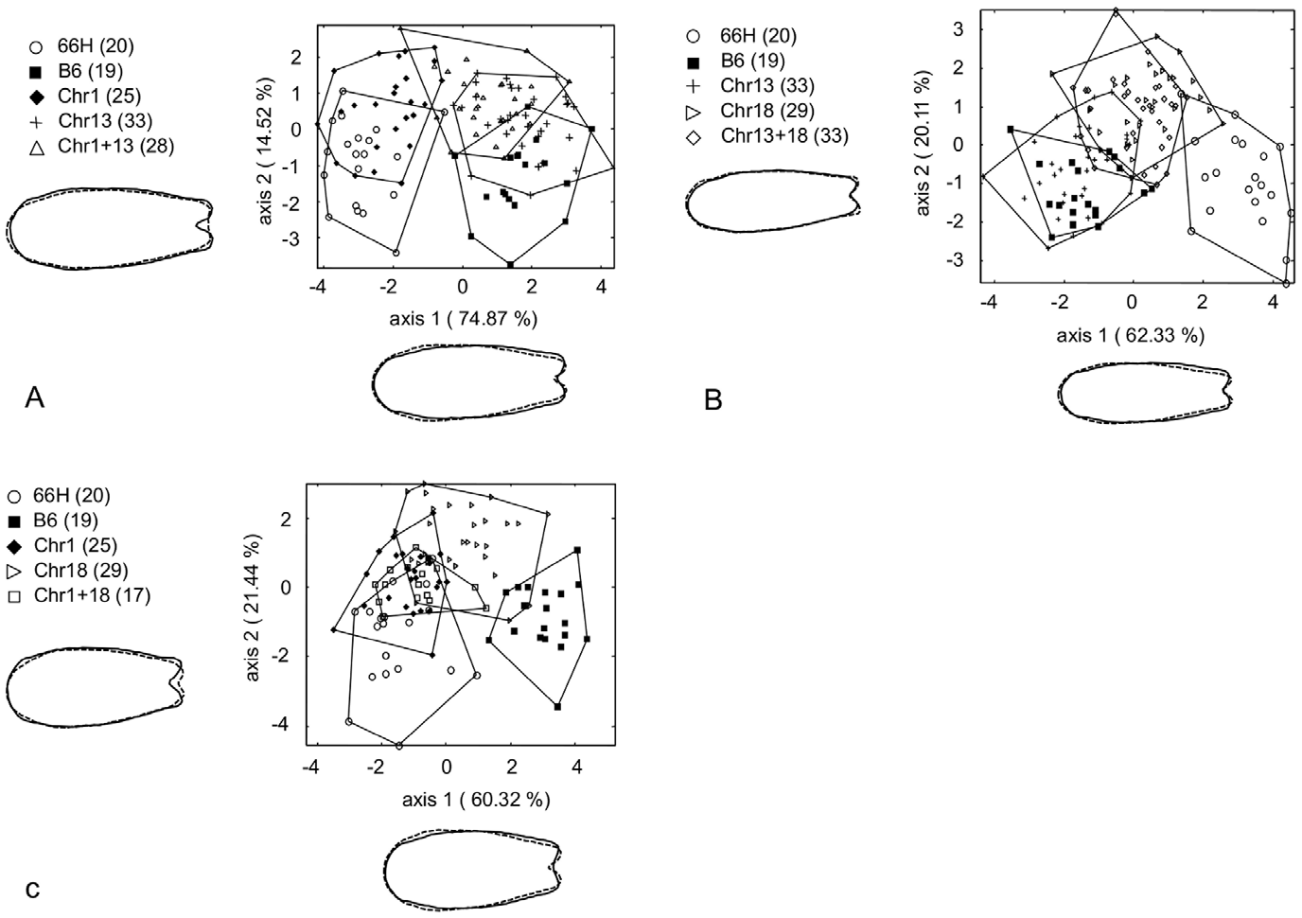
Evaluation of epistatic interactions between the three congenic fragments. Each graph represents B6, 66H, two congenic strains and the corresponding bicongenic strain. A: The first and second canonical axes for 66H, B6, Chr1, Chr13, and Chr1+13 are represented, totaling 89.4% of the total variation and showed the same phenotype between Chr13 and Chr1+13 indicating that Chr13 segment decreases the effect of Chr1 QTL. B: The first and second canonical axes for 66H, B6, Chr13, Chr18 and Crh13+18 displayed 82.44% of the total variance and exhibited no differences in nasal bone shape between Chr 18 and Chr13+18. Therefore Chr13 QTL has no effect when combined with Chr18. C: The first and second canonical axes for 66H, B6, Chr1, Chr18 and Chr1+18 represented 81.7% of the nasal bone shape variation. Chr1+Chr18 bicongenic mice show a phenotype similar to that of the Chr1 congenic and 66H. For the explanation of the shape changes, see Figure 3. No amplification of the nasal bone shape was effected.

To explore interactions between QTLs, we produced bicongenic strains by crossing pairs of congenic strains. For each pair of QTLs, LDA was performed with B6, 66H, the two congenic and the bicongenic strains (Figure 7).

Although B6-Chr13 by itself did not show any effect on nasal bone shape in comparison with B6, it was able to partially abolish the effect of B6-Chr1, as shown on Figure 7A. In fact, B6, B6-Chr13, and B6-Chr1+13 groups were largely overlapping and clearly different from the 66H and B6-Chr1 groups. Statistical inference on Mahalanobis distances provided additional confirmation. While B6-Chr1 was not quite significantly separated from 66H (T^2^ = 11.7, F*_(5,40)_* = 18.24, p = 0.06 with 1-β = 0.94), B6-Chr1+13 was clearly different from 66H (T^2^ = 36.15, F*_(5,44)_* = 72, p = 0.004) but not from B6-Chr13 (T^2^ = 7.61, F*_(5,58)_* = 10.9, p = 0.15).

On the contrary, B6-Chr13 did not significantly change the intermediate phenotype of B6-Chr18 in B6-Chr13+18 bicongenic strains (Figure 7B). This was confirmed by statistical inference on Mahalanobis distances. B6-Chr13+18 did not significantly differ from B6-Chr18 (T^2^ = 10.9, F*_(5,59)_* = 16.8, p = 0.067 with 1-β = 0.64) while it was significantly distinct from B6 (T^2^ = 20.1, F*_(5,49)_* = 35.3, p = 0.016), 66H (T^2^ = 31, F*_(5,50)_* = 60.2, p = 0.005), and B6-Chr13 (T^2^ = 19.7, F*_(5,63)_* = 34.5, p = 0.017).

Finally, the combination of Chr 1 and Chr 18 QTLs in the Chr1+18 bicongenic strain (Figure 7C) resulted in a phenotype similar to that of B6-Chr1 and of 66H, as assessed by Mahalanobis distance (T^2^ = 5.3, F*_(5,39)_* = 7, p = 0.35, and T^2^ = 11.4, F*_(5,34)_* = 17, p = 0.063 with 1-β = 0.68, respectively).

In conclusion, the Chr 1 segment (∼32 Mb) and Chr18 QTL (∼13 Mb) contains QTLs which controls the phenotypic difference between B6 and 66H. Interestingly, the effect of Chr1 QTL is abolished by the SEG-derived Chr 13 segment (∼10 Mb). The Chr 18 QTL (∼13 Mb) has a milder effect independent of Chr 13. When combined, Chr 1 and Chr 18 segments produce a phenotype similar to that of 66H and does not differ from the Chr1 segment.

### Shape Variations are not Due to Variations in Size

We observed that the size of the nasal bone was variable between B6, 66H, F1, and congenic strains (Figure S3). Nasal bone was very significantly larger in F1 mice than in any other strain (p<0.0003). Among the congenics and bicongenics, only the Chr 18 congenic strain showed a significant difference with B6 (p = 8.10^−5^).

To evaluate the influence of nasal bone size on its shape, outlines of B6, F1, congenic, and bicongenic strains were analyzed by PCA and the first 15 components were submitted to multiple linear regression against nasal bone size (calculated as the square root of shape surface). No significant multiple correlation coefficients were found, indicating that specific shape changes observed between strains did not result from variations in size of the nasal bone.

## Discussion

The aim of this study was to assess the power of IRCS for the dissection of complex traits. We focused on morphological traits because (1) the two parental strains present very distinctive gross morphology, and (2) these traits are under the control of multiple genes, a situation where IRCS are of particular interest [14]. Skull shape in mouse inbred strains has been the subject of [4] [15], [16], [17], [18]. We studied the nasal bone since, because of its position, it might be less subject to shape constraints than other parts of the skull, hence more susceptible to inter-strains variations. Furthermore, its almost flat structure allows two-dimensional shape analysis.

Morphological features such as bone shape are traits which description requires a large number of parameters to be accurate. To simplify the analysis, one may use precisely defined and reproducible landmarks [8]. However, the landmarks may be too few and their position not optimal to capture the structure complexity and variations. Simplification of the shape may result in lack of power to reveal differences. For these reasons, we used outline analysis to describe bone shape, combined with mathematical tools to reduce data complexity. We optimized parameters to ensure detailed shape description with minimal background noise.

Morphological features are typical complex traits, under both genetic control and the influence of environmental factors. There is also substantial variation between genetically identical mice raised under the same conditions, so that studies on skeletal shape on F2 or backcross populations often yield QTLs with small effects and large confidence intervals [4], [6]. Conversely, measuring shape on a group of sex-, age- and genotype-matched individuals, like recombinant inbred (RIS) or recombinant congenic strains (RCS), provides higher accuracy and power. However, genetic differences between individual strains with a set of RIS are often too large to isolate individual genes in traits with highly polygenic control, such as morphological features [9]. With one eighth of the genome segregating, even classical RCS might not offer sufficient resolution.

With IRCS, we have optimized the conditions for the detection of QTLs controlling multigenic traits in two ways. Starting from two parental strains which belong to different species, we have maximized the level of genetic and phenotypic polymorphism. For example, a first screening of a subset of this collection has identified differences for a number of traits relevant to male hypofertility and sterility [19], and embryonic lethality [20]. Moreover, because the *Mus spretus* contribution in each IRCS is, on average, as small as 1.3%, dispersed in two to three chromosomal segments with an average size of less then 15 Mb, there is a higher probability that genetic factors be isolated and underlying genes amenable to positional identification.

The present study is a successful example of this strategy. At first, a series of IRCS were phenotyped and compared with B6. At this point, the comparison with SEG was not relevant since the *Mus spretus* contribution in each strain is very limited. In addition, the entire set covers only 40% of the genome, so that part of the phenotypic differences observed between B6 and SEG are controlled by genes not polymorphic in the set. IRCS 66H was first identified as different from B6 upon macroscopic observation of the head shape in live mice. Skull observation revealed that nasal bone shape was intermediate between B6 and SEG. This difference with B6 was definitely confirmed by outline analysis.

Several strategies were used to identify which of the three chromosomal segments were controlling the nasal bone shape in 66H. An F2 cross revealed two major QTLs on chromosomes 1 and 18, seemingly acting in an additive manner. However, the most meaningful results were obtained from congenic and bicongenic strains. Our data show that the three SEG-derived regions present in 66H influence the shape of the nasal bone, either independently or in combination. The analysis of bicongenic strains revealed complex genetic interactions between loci, which were not detected in the F2 progeny. Indeed, the analysis of groups of 15 to 30 genetically identical mice was crucial to overcome weak QTL effects and substantial inter-individual variations.

The only detectable effect of the Chr 13 segment was to almost abolish the effect of the Chr 1 QTL. This observation, unexpected considering the F2 data, emphasizes that some QTLs may be missed if their detection is based on individual effects. In our case, the effect of Chr 13 was detected only in the Chr 1+13 bicongenic strain.

66H shows an intermediate phenotype between B6 and SEG, which suggests that genetic control of nasal bone shape is more complex and other genes are involved. Since only 19 out of the 55 IRCS were screened, some of these genes may be identified with a similar approach, keeping in mind that only about half of the genome is covered by this collection. We have also investigated more complex, three-dimensional, structures. Several strains have been identified, which, in comparison with B6, show features affecting specific regions of the skull [18].

In conclusion, this study demonstrates that a complex trait such as bone shape (or of other anatomical structures) can be efficiently analyzed genetically using both appropriate descriptors and genetic reference populations such as IRCS.

## Materials and Methods

### Ethics Statement

All studies on animals followed the guidelines on the ethical use of animals from the European Communities Council Directive of November 24, 1986 (86/609/EEC). All animal experiments were approved and conducted in accordance with the Institut Pasteur Biosafety Committee (Paris).

### Mice and Crosses

IRCS were developed and bred at the Institut Pasteur in Paris [14]. Detailed genetic composition is available from http://www.pasteur.fr/recherche/unites/Gfons/ircs/ircshome.htm. A F2 cross was produced by mating a 66H male with a B6 female (all IRCS carry a B6 Y chromosome). Ninety-one males were phenotyped (see below) and genotyped. An F1 male was also mated with B6 females to produce a backcross generation from which each of the three chromosomal regions was isolated as a starting point of congenic strains. Bi-congenic strains were produced by intercrossing congenic strains.

All animals were raised in the same animal room, under a 12****h:12****h light:dark cyle, and received the same food (A03/10 pellets, SAFE, Augy, France). Pups were weaned at 4 weeks of age. Up to four male mice of the same litter were grouped.

### Genotyping

Mice were genotyped using DNA prepared from tail biopsies. Microsatellite markers were genotyped according to standard PCR protocols, and using 4% agarose gels. SNP markers were genotyped by pyrosequencing according to the pyrosequencer manufacturer's recommandations (Biotage Uppsala, Sweden). The presence of the three chromosomal segments carried by strain 66H was assessed using the following markers located close to the boundaries of each segment (Figure 2). Chromosome 1: *D1Mit81* (87.599477 Mb) and *rs6259837* (119.094898 Mb); Chromosome 13: *D13Mit106* (93.838592 Mb) and *D13Mit290* (103.968912 Mb); Chromosome 18: *D18Mit23* (42.783975 Mb) and *D18Mit123* (56.096090 Mb).

### Skull Preparation and Image Acquisition

All mice analyzed for skull morphology were 60±5 days-old males. They were euthanized by CO_2_ asphyxiation. Head was separated from the body and fixed in ethanol for one week. All tissues were manually removed. The skull was then immersed in 12% sodium hypochlorite for 30 minutes, rinsed with water for 10 minutes and dried for 6 hours.

Skulls were placed on a purpose-made plastic stand for proper orientation. Dorsal side of the skull was oriented for maximum length and width, and photographed under a stereomicroscope (Nikon SMZ1500, Tochigi, Japan) using a 1.34 Mpixels digital camera (Axiocam HR, Carl Zeiss, OberKochen, Germany) and the Axiovision 3.0 software (Carl Zeiss). This resulted in a flat shape with limited loss of information.

### Outline Acquisition and Analysis

Outline was manually drawn on the digital image using the Illustrator CS software (Adobe Systems Inc., San Jose, CA) and acquired using the Tpsdig 1.4 software available from http://life.bio.sunysb.edu/morph/soft-dataacq.html. Outline points were evenly distributed along the outline. Four control points were taken as homologous landmarks for outline orientation and normalization (Figure S4).

Outlines were analyzed according to the procedure described by Baylac and Friess [21], based on the algorithm developed by Kuhl and Giardina [22], [23]. Analysis combined outline description by Fourier approximation and Procrustes superimposition. In short, a generalized Procrustes analysis (GPA) [21], [24], [25], [26] was performed using a generalized least-squares method. Control points were first centered, normalized by centroid size (square root of the sum of squared distances between the centroid location and all landmarks of an object) and rotated to minimize the overall sum of squared distances to the consensus points [25]. Transformations were based on control points and then applied to the corresponding outlines, first centered and size normalized by the square root of the surface of each outlines. Outlines were made symmetric by an adaptation for outlines of the object symmetry method [27]. The symmetrical component of the outline was taken as the average harmonic coefficients between the aligned original and reflected outlines. Outlines visualization were calculated by multivariate regresseion following the procedure described previously [26].

Outlines were analyzed by Fourier descriptors using 30 ellipses (harmonics) to accurately describe nasal bone shape. This resulted in 120 Fourier coefficients which were submitted to a principal component (PC) analysis. Taking into account 15 PCs resulted in 8-fold reduction of dimensionality.

### Statistical Analysis

For QTL mapping in the F2 cross, outlines of B6 and 66H mice were submitted to LDA. The discriminant function was applied to the first 15 PCs extracted from the Fourier coefficients of the F2 progeny to obtain a score which was used as a quantitative variable. QTL analysis was performed with R/QTL [28], using the scanone and scantwo commands. One-way and two-way ANOVA was performed using R 2.4.1 (http://www.R-project.org/).

Outlines of B6, 66H, F1, congenic, F1 generation between a congenic line and B6 and bicongenic strains were analyzed using LDA. To ascertain, the mode of inheritance and epistasis of the SEG-derived segments, separate analyses were conducted using LDA on each congenic and bicongenic lines. Classification rates were calculated by the Leave-One-Out Cross Validation procedure of the R lda function [29]. Generalized Mahalanobis distances (D^2^) between groups were calculated on all discriminant axes and outliers were included into these analyses. Groups were compared with Hotelling’s T^2^ test [30]. T^2^ statistics follows a F distribution with *t* and *n_1_+n_2_-t-1* degrees of freedom, where *t* is the total number of groups, and *n_1_* and *n_2_*, the number of individuals in the two groups under comparison. *p*-values were submitted to Bonferroni correction. The power of the Hotelling’s T^2^-test was calculated as described in [31] when *p* was in the 0.05 to 0.1 range.

Morphometric analysis and statistical analysis were performed with Matlab 6 p 5 (Mathworks Inc., Natick, MA) (M.B.) and R 2.4.1 (http://www.R-project.org/) with Rmorph library (M.B.) and MASS library with additional programming (G.B.).

## Supporting Information

Figure S1 **Assessment of the number of harmonics required to finely describe the original outline.** While 15 harmonics are sufficient for the rostral end, 30 harmonics are required to properly capture the fine features of the caudal end (notch).
(TIF)

Figure S2 **Distribution of LDA score for nasal bone shape of 66H, B6 and F2 cross.** Discriminant canonical function obtained from LDA of B6 and 66H was applied to F2 mice and used as the score represented on the X-axis.
(TIF)

Figure S3 **Variations in the size of the nasal bone in 66H, B6, their F1 hybrids, congenic, and bicongenic mice.** Size was measured as the square root of the bone surface. Error bars show s.e.m. The size of the nasal bone was consistent within strains but varied significantly among strains. It was larger in (B6×66H)F1 compared to B6 and 66H. Chr18 congenics have a significantly larger nasal bone than B6 or Chr1 congenics (p = 8.10^−5^, and p = 1.3.10^−5^, respectively).
(TIF)

Figure S4 **Dorsal view of the nasal bone.** Dashed horizontal line represents the symmetry axis. White dots show the landmarks used for outline orientation and normalization.
(TIF)
