# Supplementary figures and images for: Nasal Bone Shape Is under Complex Epistatic Genetic Control in Mouse Interspecific Recombinant Congenic Strains
Source: PLoS One. 2012 May 25;7(5):e37721. doi: 10.1371/journal.pone.0037721 (PMC3360618; doi:10.1371/journal.pone.0037721)

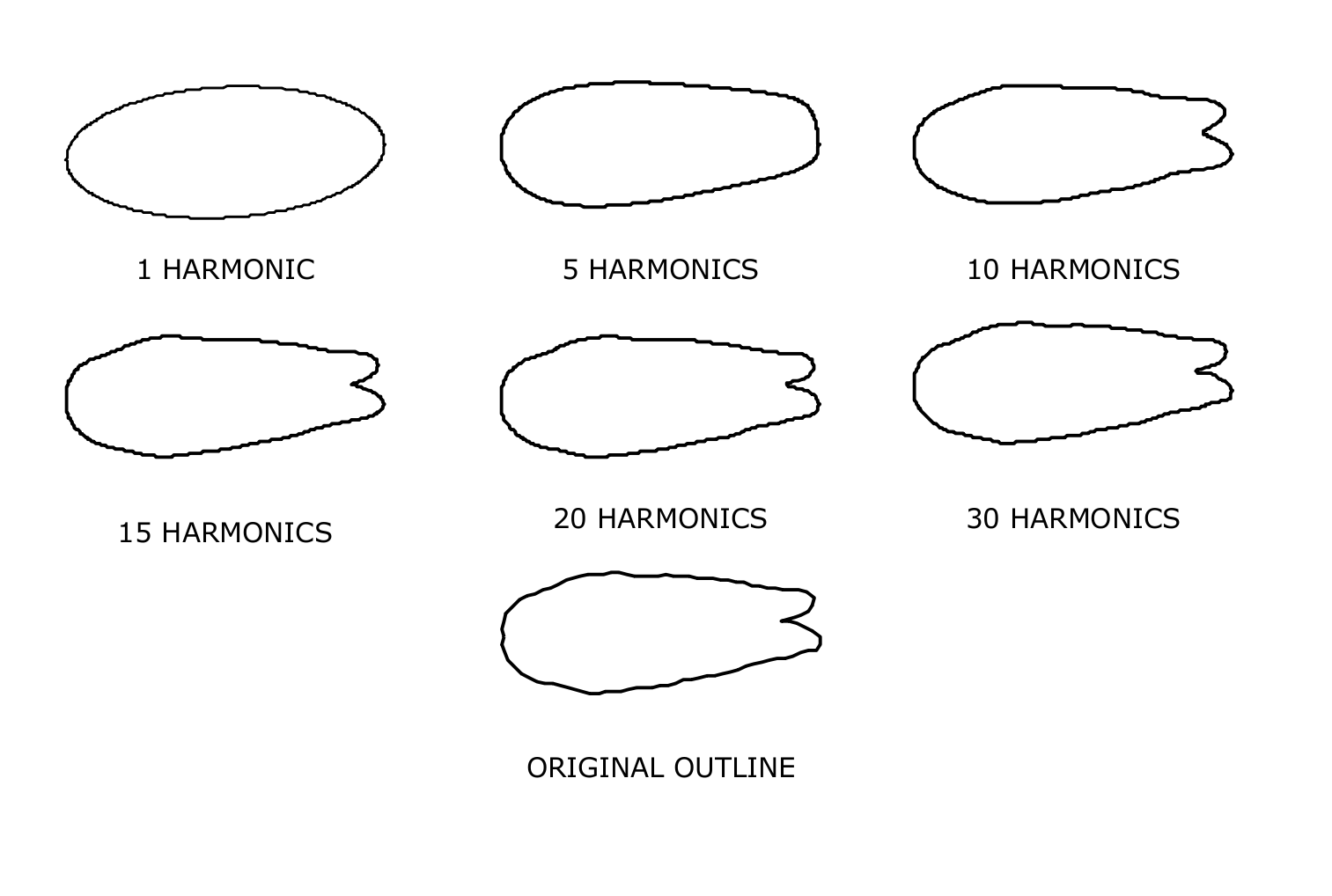

Supplement: Figure S1 — Assessment of the number of harmonics required to finely describe the original outline. While 15 harmonics are sufficient for the rostral end, 30 harmonics are required to properly capture the fine features of the caudal end (notch). (TIF) [file pone.0037721.s001.tif]

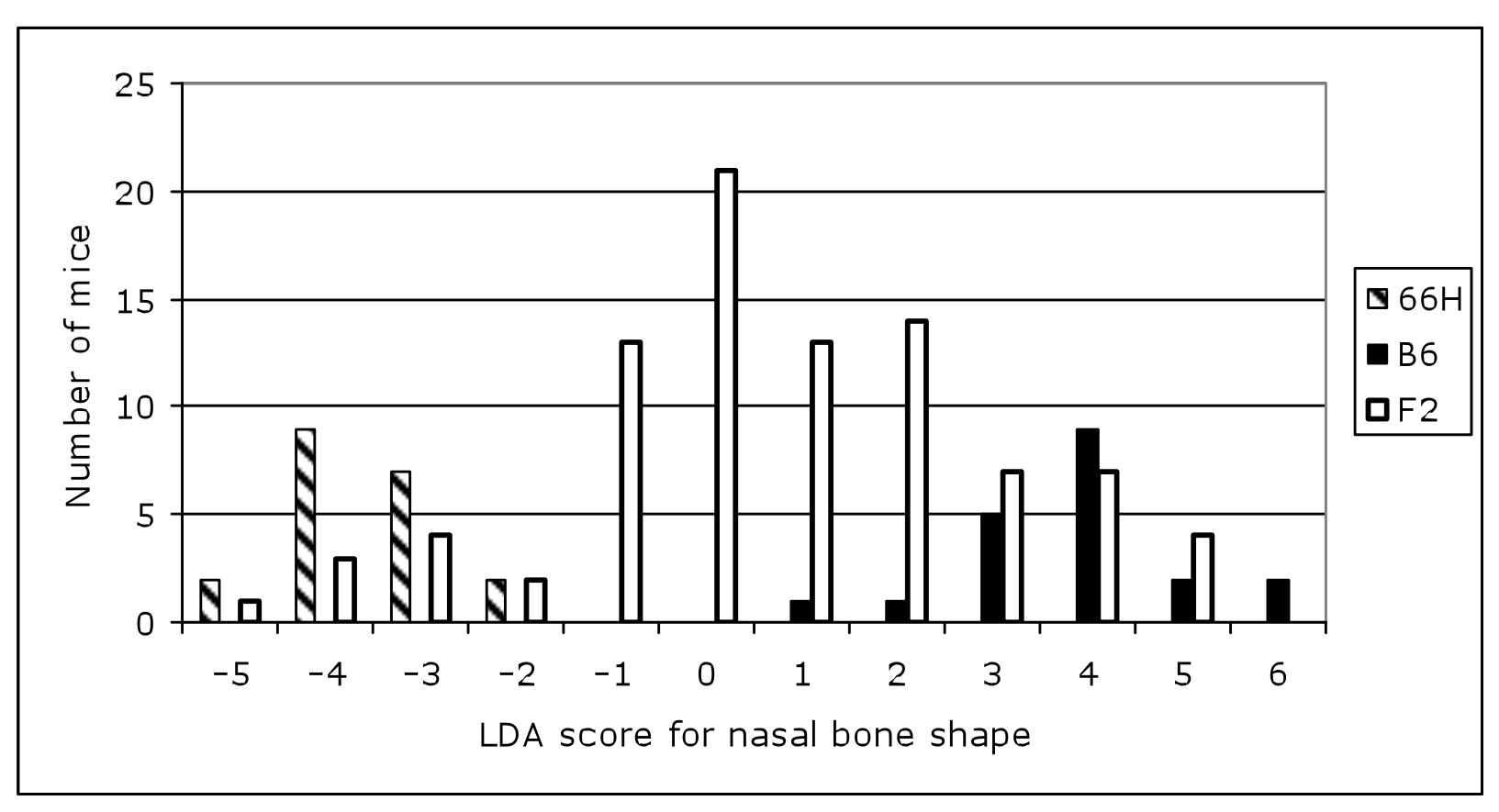

Supplement: Figure S2 — Distribution of LDA score for nasal bone shape of 66H, B6 and F2 cross. Discriminant canonical function obtained from LDA of B6 and 66H was applied to F2 mice and used as the score represented on the X-axis. (TIF) [file pone.0037721.s002.tif]

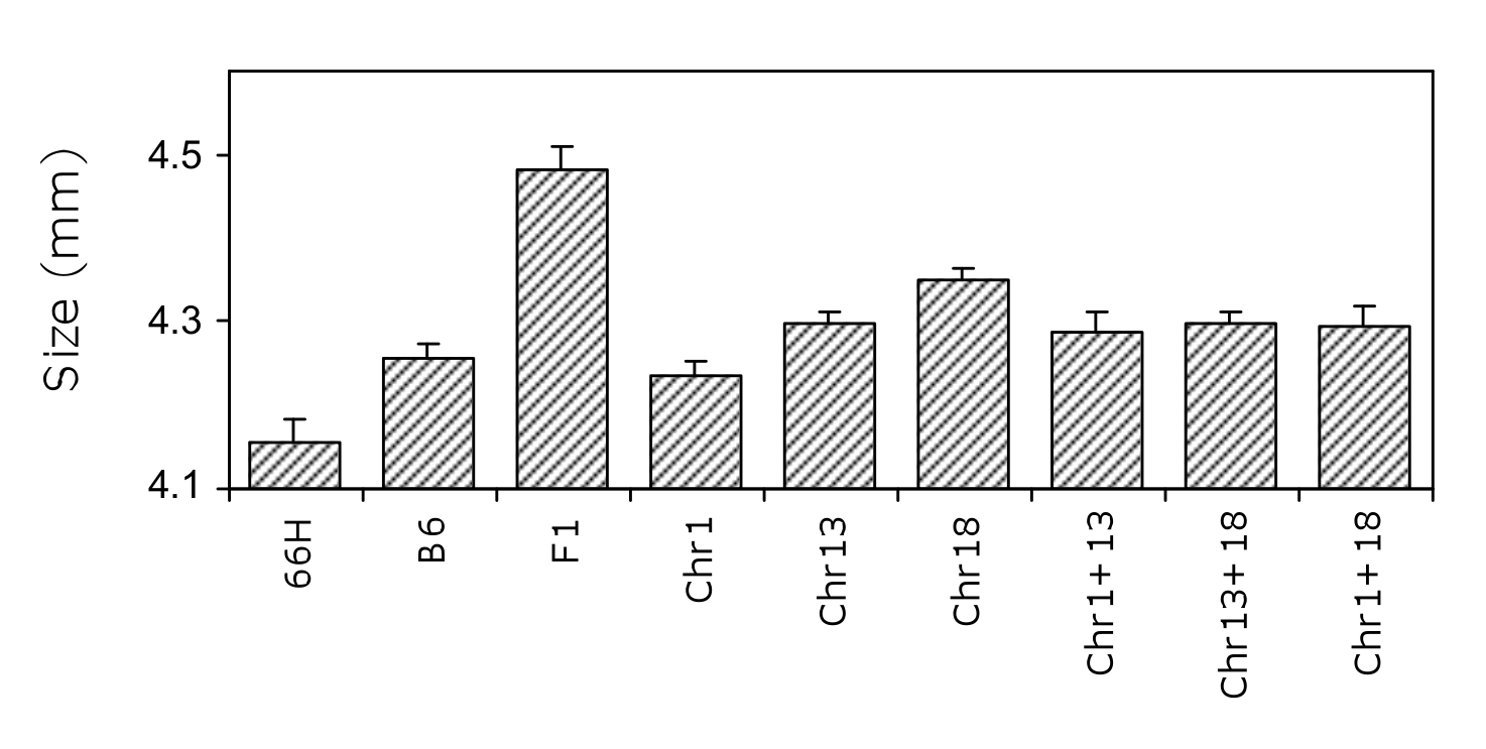

Supplement: Figure S3 — Variations in the size of the nasal bone in 66H, B6, their F1 hybrids, congenic, and bicongenic mice. Size was measured as the square root of the bone surface. Error bars show s.e.m. The size of the nasal bone was consistent within strains but varied significantly among strains. It was larger in (B6×66H)F1 compared to B6 and 66H. Chr18 congenics have a significantly larger nasal bone than B6 or Chr1 congenics (p = 8.10−5, and p = 1.3.10−5, respectively). (TIF) [file pone.0037721.s003.tif]

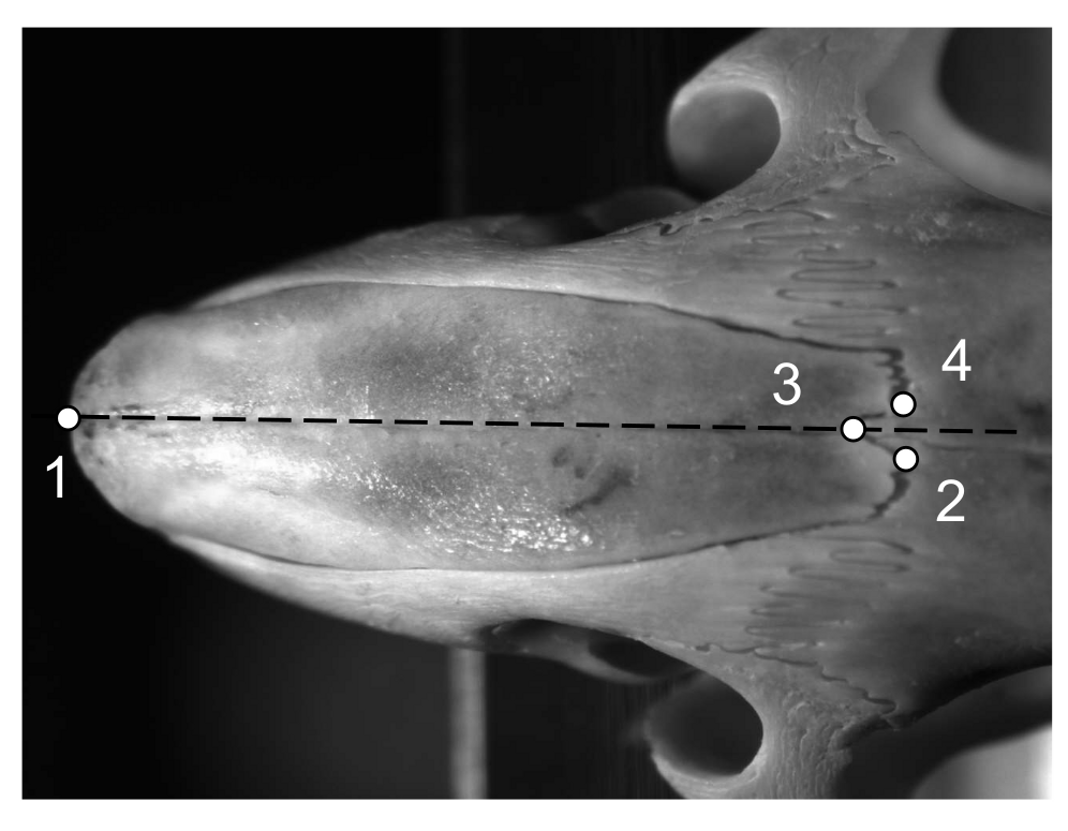

Supplement: Figure S4 — Dorsal view of the nasal bone. Dashed horizontal line represents the symmetry axis. White dots show the landmarks used for outline orientation and normalization. (TIF) [file pone.0037721.s004.tif]
